# Violence in general practice: a gendered risk?

**DOI:** 10.1111/1467-9566.12373

**Published:** 2015-10-26

**Authors:** Mary Ann Elston, Jonathan Gabe

**Affiliations:** ^1^ Centre for Criminology and SociologyRoyal HollowayUniversity of LondonUK

**Keywords:** violence, National Health Service, gender, general practice, medical profession, doctor‐patient interaction

## Abstract

This article focuses on the extent to which violence against family doctors in England is experienced in gendered terms. It draws on data from two studies: a postal survey of 1,300 general practitioners (GPs) (62% response rate) and in‐depth interviews with 26 doctors who have been assaulted or threatened; and 13 focus groups with primary care teams and 19 in‐depth interviews with GPs who had expressed an interest in the topic of violence against doctors. Most GPs, regardless of gender, reported receiving verbal abuse over the last two years, often interpreted as a consequence of declining deference to professionals, while actual physical assaults and threats were much rarer and more likely to be reported by men. Overall, women GPs were much more likely to express concern about violence and to take personal precautions, although younger male GPs working in inner‐city practices also had high levels of concern. The study shows how some aspects of family doctors’ work has been organised on gendered lines and how these contribute to the differences in experience of violence. We suggest that the increasing proportion of women among family doctors may have implications for these, often tacit, organisational routines.

## Introduction

Although there is a growing body of research and policy discussion on violence against health workers from service users (e.g. Holmes *et al*. 2012), relatively little of this focuses specifically on gender. Studies of nurses or of relatively low status carers often take for granted that these workers are predominantly female, rather than examining the implications of this (exceptions include Baines 2006). In the high status field of medicine, where women have been the minority, research on aggression has tended to focus specifically on women's experience of sexual violence and abuse (e.g. Nicolson 1997, Schneider and Phillips 1997). Men's experience of violence in the medical workplace has been largely ignored, perhaps reflecting the general emphasis in studies of workplace violence and masculinity on work strongly associated with working‐class masculinities, in which managing disorderly clients, sometimes by threatening or using violence as a ‘tool of the trade’, is a recognised part of the job (Monaghan 2002, Winlow *et al*. 2003). Here we analyse quantitative and qualitative data on client violence against both male and female doctors, specifically general medical practitioners (GPs) working in the English National Health Service (NHS). We consider how assumptions about masculinities and femininities and appropriate embodied gender performance in general practice shape the risk of violence and its management and prevention. This focus on violence illuminates some general features of gender and this form of professional work, and raises some issues about the changing gender balance in general practice. We begin with a brief account of relevant features of the organisation of NHS GPs’ work.

## The character and context of general practice work

NHS GPs provide primary medical care to almost all residents of England, usually as ‘independent contractors’ to the NHS, with a growing minority (often younger and/or part‐time doctors) employed by independent contractor GPs or other agencies. Most GPs work in group practices employing other, preponderantly female, non‐medical staff (e.g. nurses, receptionists and managers). Residents are, in theory, free to register with any local practice, subject to doctors’ consent, and to choose which doctor they see within a group practice, although reception staff may sometimes use their discretion. Most face‐to‐face consultations take place on practice premises in normal working hours, through a mixture of pre‐booked and emergency appointments.

A small (and declining) proportion of GP work occurs away from the practice. Visits may be made to housebound patients during normal working hours, but, in the context of violence, it is so‐called ‘out‐of‐hours’ (OOH) work, responding to patients’ urgent calls, that has, historically, attracted particular concern. When our data were collected, OOH services were provided through a mixture of GPs providing cover for their own patients, sharing rotas within and between practices, using commercial agencies (employing qualified GPs) and joining GP co‐operatives, owned by networks of practices, with paid managers. Currently, almost all responsibility for organising OOH work in England is the responsibility of clinical commissioning groups, although individual GPs may still choose to undertake OOH work.

## GPs at risk of violence?

Claims that NHS GPs are at increasing risk of violence have been widely made by the profession since the mid‐1990s (Elston *et al*. 2002). Such claims have been mainly based on anecdotal evidence, but there are several reasons why GPs might be at above‐average risk of violence in the workplace. Most work in ‘small businesses’ accessible to the communities they serve, sometimes in ‘high‐risk’ geographical areas. They are in constant contact with many members of the public, including the socially disadvantaged, sometimes in unfamiliar settings, such as hostels for the homeless, and at night (Budd 1999, Denney *et al*. 2008, Hopkins 2002). As for many health and personal service workers, GPs’ clients may sometimes be distressed, angry or troubled. GPs are gatekeepers, obligatory points of access to valued resources, such as prescription drugs or sickness certificates, over which there is scope for conflict with patients. GPs’ involvement with patients’ intimate and sensitive physical and emotional concerns may also give rise to adverse reactions.

This is not to suggest that violence is likely to be a frequent experience for most GPs. Patient consultations are usually routinised, orderly encounters which follow unwritten or tacit rules of conduct, including treating people politely and with respect (Stokes *et al*. 2006). But, as a cliché much used by GP's says, ‘you never know what is going to come through the door’. And it is the GPs’ job to respond to whatever does come through. Good communication skills and appropriate body language and demeanour are regarded as important aspects of professional competence, required for both effective medical care for individual patients and an orderly and efficient work flow (Roter *et al*. 2002, West 1984). Far from violence being a ‘tool of [their] trade’, GPs are expected to maintain a professional demeanour of emotional neutrality towards all their patients, and may be held to account for any failures to manage patients’ or their own emotions, such as anger and fear, within consultations.

So, considering violence in general practice invites attention to the salience of emotion and sentimental work (James 1989, Strauss *et al*. 1985) and of considerations relating to embodiment, physicality and body work of GPs (Maseide 2011). Doctors as well as patients bring their emotions and their embodied selves into professional practice, and these embodied selves are, of course gendered.

## Gender, violence and the body in general practice

Until recently, NHS general practice was predominantly men's work, albeit with a higher proportion of women than in most hospital‐based medical specialties. But the upsurge in the profession's concerns about violence coincided with a rapid increase in women GPs, reflecting women's increasing entry to UK medical schools since the 1980s. From one in five NHS GPs being female in 1985, the proportion had risen to one in three NHS GPs by 2000 (Elston 2009b). In 2013, women became the majority of all GPs working in the NHS in England (Health and Social Care Information Centre 2014). That violence might be a particular concern for women GPs was noted as their numbers began to increase. For example, a new career guide for women GPs produced in the 1990s contained a chapter specifically about ‘Combating Violence in General Practice’, that begins with an attempt to reassure: ‘There is no evidence that women doctors are more often victims than men, though women may take more precautions to reduce the risk’ (GMSC 1994: 72). Later, we will test this comment against our data. Before doing so, we elaborate further on gender, embodiment and emotion work in professional practice.

Most 20th century Anglo‐American sociology of the professions depicted the characteristic features of medical professionalism as universalistic and gender‐neutral. But by the 1990s, this assumption of gender‐neutrality was widely criticised for failing to recognise that individual, ostensibly gender‐neutral, doctors have a gender‐marked physical presence which, historically, has been mainly masculine. Conventional medical professionalism was re‐analysed as celebrating a masculinist vision of emotionally detached autonomous agents, able to control interactions on their own terms (Davies 1996). Pringle (1998) drew on Bourdieu's integrally related concepts of *habitus* (socially learned dispositions of thought, speech and conduct) and *hexis* (socially learned modes of physical deportment), to suggest that possession of a male body had been taken for granted in medicine (Bourdieu 1990, Robbins 1991).

Clinical work in general practice seems unlikely to demand frequent displays of overt hegemonic masculinity, involving great physical strength or proficiency with stereotypically masculine instruments (Connell 1987); and male GPs are enjoined to avoid any semblance of sexually inappropriate professional conduct (Bradby *et al*. 1995). Nevertheless, the entry of more women into general practice raises questions about the performance of masculinity and femininity in general practice, and about possible changes in the embodied *habitus* and *hexis* of GPs, as women become the norm. It is perhaps not entirely a coincidence that, alongside the numerical increase of women since the 1990s, a new vision of ‘medical professionalism’ has been widely promulgated by professional bodies, including the Royal College of General Practitioners, emphasising the importance of partnership with patients rather than paternalism (Elston 2009a, Pill 2010, Rosen and Dewar 2004). It seems that, at the level of patient care, all ‘new medical professionals’ are expected to perform in a less masculinist or controlling manner than their predecessors, but this raises questions about appropriate performance when patients are disruptive and threatening.

This article focuses on female and male doctors’ experience and management of violence in their daily work in order to shed light on gendered *hexis* and visions of professionalism in contemporary general practice. Moreover, we suggest that, if violence is a useful lens through which one can examine gender in general practice, general practice may also be an appropriate locale in which to examine gender and violence. Feminist approaches in criminology conceptualise men's violence, particularly sexual violence, as a means by which men exercise power over women, especially but not only in the private sphere in the form of domestic and sexual violence (Kelly and Radford 1996, Stanko 1990). In general practice, female doctors may see male patients and *vice versa*, and patients’ private matters may come under professional scrutiny. Examining violence may, therefore, shed light on the management of the gendered doctors’ body and of sexuality in patient‐professional relationships, and make a contribution to the study of gendered embodiment at work (Crawley *et al*. 2008, Wolkowitz 2006).

## Methods

The data reported here are mainly drawn from a UK Economic and Social Research Council (ESRC) funded study of Violence against Professionals in the Community. A postal survey was sent to a one in three sample of GP principals in the former NHS South Thames region and 62 per cent responded (n = 697). Except for slight under‐representation of older, overseas‐trained doctors, respondents were representative, in known socio‐demographic characteristics, of fully‐trained GPs in England. Women were 37 per cent of all respondents, and were more likely than men to be less than 40 years old (38% compared to 25%), and to be working on less than full‐time contracts (37% compared to only 5% of men). Younger doctors of both sexes were more likely to be working in inner city or deprived estate areas than older doctors, with no overall gender differences in locality after controlling for age.

Drawing on previous research (Hobbs 1994, Naish and Stevens 1998) and policy documents (Health and Safety Commission 1997), and following feminist researchers’ critique of much conventional criminology (e.g. Kelly and Radford 1996), our survey asked about not only physical violence but any incidents involving verbal abuse, threats or assaults related to work, including sexual harassment and abuse, in the previous two years. We use ‘*violence’* (italicised) as the collective term for these three types of transgressive behaviour. Information about the context for the most recent incidents of *violence*, practice organisation and environment, and GPs’ biographical background was also collected. We also gathered data on the extent to which GPs were fearful of violence, how they managed the risk of violence and tried to minimise the possible harm. Data were analysed using SPSS for Windows (IBM, Armonk, NY).

We then conducted follow‐up interviews with a sub‐sample of 26 respondents, 17 male and nine female, who had reported at least one incident of physical assault or threat in the postal survey, over‐recruiting women and minority ethnic group GPs in order to ensure representation of their views. Reflecting the incidence of physical assaults and threats found in the survey, most interviewees practised in inner city or other relatively socially disadvantaged areas. The interview topic guide covered, amongst other things, the specific incidents that had triggered selection for interview (and other incidents that were reported in interview), the doctors’ work context, fear and approaches to preventing and managing violence. Interviews were undertaken at the doctor's practice and typically lasted about 1.5 hours. Recordings were fully transcribed, and analysed using ATLASti (ATLAS.ti Scientific Software Development GmbH, Berlin), identifying themes from the topic guide, and developing new categories that emerged from the data.

Further data were obtained from a second qualitative study of managing and preventing violence in general practice, undertaken shortly after completion of the ESRC study. Following purposive sampling of London area practices expressing interest in the topic, we conducted 13 focus groups and 19 further in‐depth interviews with GPs (13 men and 14 women) and other primary care staff, including receptionists and practice managers. Themes emerging from interviews for the ESRC study and pilot interviews for this study informed the focus group topic guide. This guide focused on the meaning and experience of violence and strategies for preventing violent incidents from occurring. Vignettes of stylised ‘problem patients’ and what might be viewed as ‘less than ideal practice’ were also used to reduce the risks of sensitive disclosure in group contexts and to provide standardised examples of incidents across groups. The interview topic guide explored similar issues around the meaning and experience of violence and incident prevention, as well as training needed to manage and reduce violence. Data from this study were analysed after the ESRC study was completed. When we compared the qualitative data from the two studies we were struck by the similarities of the views and experiences expressed by GPs in these studies. Such similarities suggest that our respondents were typical of GPs practising in urban South‐East England.^1^

## Findings

### Experience of violence

Summary survey data on the incidence of *violence* are shown in Table 1. Taking the three types of transgressive behaviour together, almost four out of five GPs reported at least one specific incident in the previous two years (although very few reported more than ‘two or three’). There was no gender difference in this overall risk. For both females and males, the vast majority of incidents were of verbal abuse. Although physical assaults and threats were much rarer, male GPs were significantly more likely than females to report at least one of these types of incident. Younger (aged less than 40 years) male doctors in inner city practices were the most likely sub‐group to report any form of *violence*, significantly more so than their older male peers.

**Table 1:** General practitioners’ reports of incidents of “violence” in previous two years and of fear by gender

	% of all Male GPs	% of all Female GPs	N	Sig (χ^2^)
At least one incident (assault, threat or verbal abuse)	78	78	674	n.s.
Physical assault	13	7	674	p<.02
Threat of harm	33	18	674	p<.001
Verbal abuse	74	78	666	n.s.
Afraid of becoming a victim of violence	60	76	673	p<.001

Approximately 80 per cent of ‘most recent’ incidents of *violence* were attributed to patients or their relatives, occurring on practice premises, with no statistically significant gender differences, although incidents were slightly more likely to have occurred away from practice premises for men than for women. Three men but no women reported a physical assault while on a night call. Being pushed or shoved was the most common form of physical assault. Four doctors, three of them female, reported being indecently or sexually assaulted by a patient in the past two years. In 82 per cent of all ‘most recent’ physical assaults, the assailant was male, but there was a significant difference according to assaulted GPs’ gender. For males, 89 per cent of assailants were male whereas for women, only just over half, 56 per cent, were (χ^2^: p < 0.001).

### Fear and the impact of violence

As well as occasionally experiencing *violence*, most survey respondents (male and female) also reported sometimes feeling ‘afraid of becoming a victim of violence’ (Table 1). Overall, women were, however, significantly more likely to report this than men. Controlling for ethnicity and type of contract (full time or part time) did not affect this gender difference. Among women, the percentage of respondents reporting feeling afraid did not vary significantly by age group, location of practice or by experience of *violence*, but it did among men. Male GPs aged less than 40 years were almost as likely as all females to report feeling afraid (70% compared to 76%). Practice premises were the most frequently cited context for fear, but patients’ homes and ‘travelling to see patients’ also figured prominently for both women and men, particularly at night.

Given the sampling methods employed, it is unsurprising that almost all interview and focus group participants reported some concern about violence, or being fearful on occasions. But participants (male and female) were usually keen to emphasise that violence was a ‘background rather than a constant concern’. The GPs often drew a distinction between what they termed ‘violence’ (that is, actual or threatened physical assaults), reported as very rare, and somewhat more frequent ‘aggression’ (verbal abuse), although receptionists were consistently said to be at higher risk of the latter than doctors. No GPs described themselves as working in an atmosphere of regular intimidation or high risk. Almost every interview contained references to circumstances and characteristics that made GPs normally feel safe, or to the rarity of frightening occasions and contexts. As one woman GP put it: ‘You can't do the job if you're afraid all the time’: implying that constant fear is incompatible with coping with the workload and attending properly to patients’ welfare, and with accomplishing ‘profession’, in the sense of displaying appropriate control and competence (West 1984).

Experiences of *violence* were not always described as being of personal or professional significance. In the survey, specific incidents were mostly rated as having no lasting impact on GPs’ mental or physical health or professional practice. Few assaults had resulted in any physical injury. Similarly, in many interviews, GPs, both male and female, downplayed the impact of any violence they had experienced, employing a ‘minimising discourse’ (Kelly and Radford 1996): telling stories about incidents in which ‘I was lucky, it could have been much worse’, or stressing that there were much worse things that could happen to a doctor. Having been knocked to the ground by a highly disturbed patient was, according to one female GP, insignificant compared with a malpractice allegation she was currently facing. In the relatively public setting of focus groups, both men and women sometimes recounted ‘war stories’ about successfully coping with danger, displaying their professionalism. Some incidents were, however, far from trivial. Two older male doctors had taken early retirement as a direct consequence of being assaulted. Moreover, alongside the minimising discourse were many references to fear, vulnerability and the emotional impact of violence and aggression, both in general and in relation to specific incidents.

A recurrent theme in the qualitative data from GPs and their co‐workers, both female and male, was that violence and aggression were, or were likely to be, of greater concern to women than to men. For example, one woman GP commented that she had tried, unsuccessfully, to get her male partners to take the issue seriously before, she noted ironically, she was assaulted. But this did not mean the qualitative data indicated a simple gender division into concerned, fearful women and confident, nonchalant men, any more than the quantitative data did. There were some gender differences. While both men and women GPs spoke of ‘fear’ and of being ‘vulnerable’, the terms used often differed: men were much more likely to use words like ‘wary’ and being ‘concerned’, whereas women were more likely to speak of being ‘terrified’, ‘very frightened’, ‘trembly’ or ‘really scared’, by verbal abuse as well as threats and physical assault. For example:

Only one patient with me who got increasingly more agitated and I really felt, um, he was going to do something because he was violent, verbally so abusive, that I felt very frightened. (Dr R. f) 2

Such differences in terms and tone used may reflect general gendered patterns of speaking about fear and danger, rather than differences in experience of incidents. But there was sometimes a particular emotional intensity to women's accounts of what we termed ‘menacing incidents’ in which ‘nothing really happened’ (Kelly and Radford 1996) but which were very frightening at the time: incidents that are hard to capture in structured questionnaires (O'Beirne *et al*. 2003), and are unlikely to be reported as violence in organisational records. All female GP interviewees and focus group participants mentioned at least one such incident, whereas only half of men did. The majority of these incidents involved harassment from male patients or members of the public, a point we return to below:

The only single, seriously unpleasant incident in my career was when I'd gone to a big block of flats … there was some men of about 18, 20, 22, outside the lift door … they sort of blocked my exit and I tried to come out and they sort of jostled me and they were smiling. It was humorous but it was a controlled sort of smiling. (Dr W. f)

As Table 1 shows, however, sometimes feeling afraid was not confined to women. Indeed, some male interviewees commented on the impact being physically assaulted had had on their professional sense of self and on their male embodied *habitus* (and the significance of ‘war stories’):

I think that's one of the reasons why it [assault] affected me as much as it did, in that, um, I'd been a sportsman, I was fairly, you know, physically active, felt that I was, you know, stronger than average, didn't really feel threatened. I mean, I had travelled all over the world with not ever being threatened. Um, it was the first time I'd ever been beaten in a fight since I was at school, and I always had the physical self‐confidence which was then very threatened. It suddenly made me feel very old, you know. Um … it's just, you know, aware of your vulnerability. (Dr G: m)

This linking, by Dr G, of confidence (or its absence) to physical prowess and strength, exemplifies a recurrent theme in our qualitative data: the significance of GPs’ (and patients’) gendered, embodied *hexis*. If violence was regarded as a particular concern for women, this was because women were perceived to be more vulnerable to harm if attacked because of being physically weaker, or to sexual violence and harassment. For example, this, GP described the type of patients who made her, as an ‘average‐sized woman’, feel nervous:

I think they are male. They are. And they are frightening ’cos they're big, and we've got one bloke and Cathy [another GP in practice] actually said [to patient] … ‘You're enormous, you're just terrifying and you terrify us and you terrify the receptionists, and don't do it’. ’cos he'd come in and he was young and muscular and he's frightening. (Dr T: f)

Some of the male GPs explicitly drew attention to their masculine physical embodiment as a source of confidence: ‘I think there is a male issue in a sense that, you know, I'm not obviously a five stone weakling’ (Dr A: m). This was sometimes contrasted with female colleagues’ perceived vulnerability, for example, when aggressive patients phoned for out‐of‐hours home visits:

[They say] get your effing arse down here. It's ok for me. I am a six‐foot, bald‐headed bloke, and people don't mess with me generally. But there are three female GPs here and that would be exceedingly, er, yes they have to face that. (Dr M: m)

Some women GPs, however, saw their gendered embodiment as affording them protection:

I suppose because I'm a small person I have an advantage in a way, because I think, for even the most angry man, he still does think about you being a small woman. I may be, that may be a fantasy, but I think there is an element of … of a different approach, if you're a, you know, a female. (Dr Z: f)

And, conversely, this male GP was not unique in seeing his masculine *habitus* and *hexis* as potentially counter‐productive:

I am fairly conscious of my own body language and the effect that can have. Um, I think in the past I have appeared to people to be this, you know, tall white man who speaks as though he's been to university, you know. Rather a forbidding sort of figure. And I come across when I'm under pressure as appearing to be rather arrogant, and I'm very conscious of that and try to counteract it. Um, I don't always succeed. (Dr H: m)

### Sexual assault and harassment

Although the vast majority of transgressive incidents reported by GPs were not explicitly categorised as sexual violence or harassment, there were, as already indicated, some specific experiences, notably ‘menacing’ incidents, which clearly fell into this category. And general concern about sexual harassment from men was often expressed by and with respect to, female GPs and staff. Several women GPs said that they were automatically wary with male patients who were not part of their normal caseload. One male patient had left this GP feeling very vulnerable, not least because he was seen as challenging the boundary between public and private by harassing her at home:

I remember it with Colin, this man I was telling you about, the tall guy who … I suddenly became, when things got more and more difficult, very anxious about him finding out where I lived, you know … would pitch up and sort of hang around outside the house. That sort of vulnerability is, I think, very … increasing for women. I mean maybe that's a generalisation but certainly increasing for people who work fairly near their practices and have a very personal profile. People do know all about you, if you like, as a GP. (Dr F: f)

The ‘mean’ streets and ‘rough’ estates in which they made home visits, were sometimes seen as particularly dangerous places for and by women GPs. One reported that, when she had first come to work in her current practice, she had been warned by young women on the local estate that she should not visit it on her own (warnings that she said she had ignored, without experiencing any problems). In short, women GPs often talked as women, about women's taken‐for‐granted ordinary fears and ‘everyday dangers’ (Stanko 1990), dangers which were sometimes heightened when professional work brought them into risky situations. But, as with violence in general, incidents were often minimised or, occasionally, laughed off, as in this account of an attempt by a patient to assert himself in a sexualised way against a woman GP:

He [drunk male] came in effing and blinding and giving aggro to the girls [receptionists] and I went out and said ‘Look, you will not be seen drunk’. This guy walks right round the building until he identified my room and then he stood there, unzipped himself and pissed on my window. I didn't know whether to laugh or get angry and I thought ‘Oh gosh’ [words indistinct – laughing]. (Dr R: f)

Some women GPs explicitly suggested that their professional standing protected them. For example, one contrasted the courteous response received when asking directions on a night call from customers in a ‘so‐called rough pub’ on a notorious estate, with being subjected to the ‘most lecherous, disgusting language from all those supposedly City professional people’ when she entered a city bar off‐duty looking for a friend (DI.2.GP2: f). Another described the protection she experienced on a local ‘high‐crime estate’, as a female professional:

You will see one of those horrible teenage boys that your previous patient has told you has beaten Grandma up and you are walking the estate and they are going, ‘Hi doc, how you doing’ and they are looking out for you because you have managed to maintain your civility and you have not been in a situation of having any confrontations with them or whatever. They are looking out for you. They know who you are and they will make sure you are OK. (GP7: f)

Risks associated with men's violence to women were not confined to women GPs. Several male GPs reported being threatened or physically assaulted by the violent partners of female patients. But women GPs also often spontaneously described their male colleagues as being at risk from female patients, explicitly juxtaposing this with the sexualised danger they themselves might face from male patients:

[O]n the whole women [patients] aren't as aggressively sexual as men. But there is this sexual, predatory aggression that a female patient can show to a man doctor … The men [GPs] are actually quite um … anxious about the female aggressive patient because of the possible sexual innuendoes … But there are patients, male patients that make female flesh creep. [Other participants indicate agreement] (FG12: GP3: all female group)

Dr Z commented that, among the medical students she taught, ‘we have some gorgeous young men’ whom she made think through what they would do in potentially compromising situations with female patients. She immediately added that her own trainer had protected her from the most frightening aspects of on‐call work in her early days. Only one male doctor referred spontaneously to the danger posed to men by female patients: a single‐handed doctor who commented that, for him, being mugged when out on a call, was of less consequence than he imagined being accused of sexual misconduct with a female patient would be.

### Reducing risk, minimising harm and managing trouble

If women GPs are more concerned about violence, this could affect their working practices and arrangements in ways that might affect their risk of *violence*. In the questionnaire, GPs were asked whether they ever took various personal measures to reduce risk and minimise harm. As Table 2 shows, only a minority reported adopting any of these measures, but women were consistently more likely to do so than men. The difference was least for being accompanied when seeing certain patients, which may reflect men's use of ‘chaperones’ when examining women patients. Women were fifty per cent more likely than men to say that they usually left their visit schedule with someone for safety reasons. Although only a quarter of women said they sometimes carried personal alarms, they were five times more likely to do so than men.

**Table 2:** Personal violence prevention measures

	Percentage reporting ‘never’ undertaking measure for reducing risk of violence			
	M	F	N	Significance χ^2^
Check patient notes in advance	52	37	620	p<.001
Leave door ajar when seeing certain patients	80	67	604	p<.001
Accompanied when seeing certain patients	70	64	610	p<.05
Accompanied on visits to certain patients	77	65	610	p<.005
Leaving visit schedule with someone	48	25	618	p<.001
Carry personal alarm	95	77	583	p<.001
Attended self‐defence course	94	85	590	p<.005

Our qualitative data showed most GPs, women and men, were strongly opposed to so‐called ‘fortress medicine’ – GPs’ retreating behind significant physical, organisational or procedural barriers for protection – as at odds with professional values and likely to be self‐defeating, provoking rather than reducing aggression. Many were ambivalent about the practicability and appropriateness of some standard safety recommendations, for example in relation to consulting room layout, ‘You actually set it [consulting room] up for sharing and caring [mildly ironic emphasis] … You know, to be able to say “There, there, rather than Go away”’ (GP2: f). In general, GPs emphasised the importance of professionalism and good communication skills (i.e. sentimental work) for reducing risk and harm. But there were indications that women GPs might sometimes take or be afforded special protection measures, although not all of our female respondents would have accepted measures such as the following as necessary or appropriate:

I think the lady doctors are OK … they've got an alarm somewhere if they know where it is and the nurses have an alarm. (Dr O: m)

More general gender differences in practice organisation and GPs’ work patterns emerged in our qualitative data, however, which shaped the risk of different forms of violence. One such difference is in the demography of patient caseload. For all NHS GPs, but especially female ones, most consultations are likely to be with women of all ages or children (Brooks 1998). Young men have the lowest consulting rates of any demographic category (Rowland and Moser 2002). Our survey found that in 82 per cent of ‘most recent assaults’ the assailants were men, usually estimated to be aged 18–25 years. If, when they do consult, young men are more likely to see male GPs, this may partly account for our finding that 89 per cent of these assaults were male on male. Younger male doctors in inner cities, who reported the highest rate of *violent* incidents may have caseloads which include an over‐representation of young men. In contrast, as noted earlier, female interviewees commented that being consulted by unfamiliar young men was unusual (and sometimes disquieting). Our data indicate that, as well as patient's choosing to see a doctor of their own sex, some patients may be steered by receptionists away from women doctors, with their apparently more vulnerable bodies, for safety reasons:

I think we tend to be protective of Dr Ahmad because she's female and there's not a lot of her (FG6: receptionist (f)).

Explicit practice policies that particular male patients should not be seen by women doctors were sometimes reported, as in one practice for a patient who had served a prison sentence for murder. In another, a woman GP found, when she joined the practice, some male patients’ files were marked ‘Not to be seen by a woman’, a measure which she dismissed as unnecessary. Many participants spoke about patient ‘swaps’: sometimes explicit, sometimes tacit, reciprocal responses to the different risks faced by women and men GPs. Male GPs took on greater responsibility for ‘dangerous’ male patients. Women GPs took more responsibility for women patients’ intimate problems.

Clearly, such reciprocity was far from universal. Not all dangerous male patients were steered away from women doctors, as some in our study had been assaulted by men. One woman described being rescued from a frightening consultation with a male patient by a male partner who told her that she should never have seen this patient, known to be violent and abusive to women, although no steps had been taken to prevent this happening and our survey found that one in five perpetrators of physical assaults were female, almost all attacking women GPs.

Close analysis of qualitative data on the context of incidents revealed further ways in which assumptions about doctors’ gendered *hexis* might shape work practices. Nearly all the specific threats and physical assaults on women doctors, whether by men or women, arose during consultations and involved acute mental illness or a dispute about drugs (or both). In comparison, the assaults and threats that male GPs described were more varied, more often alcohol related, and more often occurred outside the consulting room, for example, in the practice waiting room. GPs, receptionists and practice managers repeatedly told us that, if there was trouble on practice premises, male GPs, if available, were called on first to sort it out. Some male GPs indicated that they might intimate the possibility of using, or, very, very occasionally, actually use their physical strength with drunk and abusive patients:

Interviewer Why are you called in when there's trouble?

Dr J (m) ‘Because I used to play rugby … It's only common sense.

We tried cajoling … he wouldn't go. I said, ‘Call the police’ and I can't remember exactly what happened. I remember helping him out, anyway. He was a bit drunk, pushing him in the right direction anyway. I mean he was pushing back a bit, but nothing drastic. He wouldn't lash out. (Dr O: m)

Although female GPs sometimes acted as ‘professionals taking charge’ of disorder on their premises, they only reported using verbal persuasion, which could mean occasionally having to rely on male patients to help out:

I think it's not realistic to expect female staff to hold on to a male assailant. If [male GP] is not around, we're at the mercy of men in the waiting room really. Them helping. I don't think female staff should take on a male role. (Dr W: f).

The only example in our dataset of a woman GP apparently initiating physical contact in a dispute with a male patient revealed the unusualness of such action: ‘I seized him by the lapels, and he was so surprised, and I was so surprised’ that the tension was defused.

Our data, therefore, indicate that male GPs are more likely than their female peers to be consulted by those allegedly responsible for most of the assaults reported in the survey – that is, young men. Moreover, assumptions about gendered *hexis* led to male GPs being more likely than females to be called on to sort out disorder on practice premises. As a result, male GPs are more likely to report being physically assaulted or threatened. At the same time, however, a minority of GPs saw these assumptions as ill‐founded or even counter‐productive, a view concisely expressed by one male GP: ‘what you need to deal with violence’ is a ‘very small nurse’ (Dr1: m). It is conceivable that women GPs might, on average, be more effective at reducing tension and deflecting aggression, perhaps by using less forceful body language or communicative styles. But, as already shown, women GPs were no less likely to report being verbally abused than men. Having a mainly female caseload did not protect women from this form of *violence*. According to this GP:

Ten or fifteen years ago, it [verbal abuse] was always the men and now it's increasingly the women. (Dr X: f)

Turning to the specific risks of and concern about OOH work, at the time of the survey, women GPs, particularly younger ones, were less likely than men to be doing such work at all. This was mainly because women were more likely to be on part‐time contracts, to have responsibility for small children, often with a doctor partner himself doing night work, or to be working in practices which contracted out all OOH work. The majority of GPs of both sexes, however, had current or previous experience of OOH work. In interviews and focus groups, they described precautions they all routinely took against, for example, being mugged for prescription pads or controlled drugs. But women also usually spoke of adopting, in the words of one, ‘just a sort of street‐wise, female, inner‐city strategy’. This might include always telling someone where they were going, even, on occasions, taking their male partners with them. Some, however, commented that, before they had husbands and children, they had been what seemed, with hindsight, heedless of possible dangers: ‘no‐one would have had a clue where I was if anything had happened’. No man reported ever taking a wife or girlfriend on a home visit.

Gender considerations shaped both the workforce and work organisation in OOH provision. For example, according to a co‐operative manager, the GPs most keen to do lots of shifts were young men who had recently become fathers and wanted to earn extra money. When women did work for co‐operatives, they were much more likely to do only ‘base work’ rather than home visits: that is, giving phone advice, often from their own homes, or working in a primary care centre, where there would usually be other staff present. Some, however, did do car work, and our qualitative data include extensive discussions of the actual or potential role of (presumed male) drivers in risk reduction, particularly for women. Not all were in favour of women (or men) being accompanied routinely by their drivers into patients’ homes. This GP thought that the initial adoption of such a policy for women GPs in her co‐operative was completely ‘over the top’:

I thought it was just amazing, the very first visit I did … I felt as if I had heavies, you arrived in a car which was about twice the size of any car I had ever driven and there was this man who was about twice as big as me who held on to my bag. And I felt I was like a rent collector or something on this big huge estate. (DI2.GP1: f)

The policy was subsequently changed to GPs of both sexes using their discretion about being accompanied.

## Gender, violence and doing the ‘same’ job?

Our study shows that violence is a ‘background’ concern for both women and men GPs, particularly those working in inner cities, although women are more likely to express concern and report experiences of being afraid. The great majority of both women and men mentioned some incidents of verbal abuse. Threats and physical assaults were much rarer, but more likely to be experienced by men than by women. Women GPs’ lower risk overall of threats and assaults may partly reflect their greater likelihood of adopting specific personal risk reduction measures compared to their male colleagues. But it is probably more related to differences in patient caseload and working arrangements, which were informed by ‘commonsense’ assumptions about gendered embodiment: that is, concern, on the one hand, about feminine vulnerability and masculine strength and control in relation to physical and sexual violence from men – and, on the other hand, about men's assumed vulnerability to allegations of sexual abuse from women patients. Here, then, male and female doctors are drawing on previously formed ways of doing gender and sexuality to construct strategies to manage violence in particular settings (Messerschmidt 2012). There were also, however, accounts that explicitly inverted the gender emphasis (Crawley *et al*. 2008), suggesting that feminine vulnerability, women's small stature or lack of physical strength could afford protection against violence. And a few explicitly suggested that a masculinist professional *hexis* was no longer appropriate, being likely to generate antagonism rather than deference from patients, or not good for doctors – of either sex.

Increased entry of women has rendered violence and its management more visible within general practice. Any tacit presumption that GPs are normally ‘big blokes’ able to take care of themselves is clearly no longer plausible. But our study found women and men were often not doing exactly the ‘same job’. The general tendency for women doctors’ time to be pre‐occupied with women and children, GPs’ ‘swapping’ risky females for dangerous male patients and the gender division of labour in handling disruption on practice premises and in out‐of‐hours work, contributed to the lower overall incidence of direct threats and physical assault experienced by women GPs in our survey. But these differences gave rise to two general concerns. The first, raised explicitly by some participants, was about equity and professional equality. Some women doctors were concerned that ‘doing femininity’ meant that women doctors were not giving their patients ‘the same’ professional service or were not living up to the ideal of the competent doctor in control, and that using ‘emphasised femininity’ (Crawley *et al*. 2008: 47) by demanding special consideration for women, might be resented by male colleagues.

The second concern is that many of the factors we identified as, in practice, reducing women GPs’ risk of being assaulted, relied on the availability of male colleagues. When we collected our data, women were just under 40 per cent of the GP workforce. Given recent recruitment and retirement trends leading to women becoming the majority of NHS GPs, some of these often tacit strategies for reducing risk may be becoming more problematic. For example, women GPs’ caseloads will, on average, see an increase in male patients, which may raise more concern over sexual harassment and means to avoid it. More women GPs will be becoming the senior clinicians in their workplaces. Changes in the gender balance in general practice, and in organisational arrangements for providing NHS primary care, may lead to changes in the reported risks and ways of managing *violence* compared to the detailed findings reported here. Recent training strategies in the NHS emphasise what might be seen as non‐gendered or even feminine approaches to risk reduction, using both verbal skills and bodily posture (such as remaining seated) and withdrawing from the situation wherever possible to de‐escalate risk of violence (NHS Protects 2013, NHS Security Management Service 2009). But such changes would not diminish the value of focusing on how assumptions about masculinity and femininity, gendered embodiment and professional *hexis* influence the quotidian organisation of GPs’ work and produce gendered patterns of risk.

## Notes

1 In accordance with university departmental ethical review approval, all participants in both studies were provided with information sheets about the study. Interviewees and focus group participants were given further sources of advice and counselling. Informed consent and copyright declaration forms were completed.

2 Extracts from transcripts are identified as follows: ESRC interviewees are identified by letter, pilot study interviewees by number. ‘DI2’ refers to interviews with 2 participants, and ‘FGn’ to focus groups with n participants.
